# Surgical Resection of a Metastatic Lung Tumor with Polypoid Extension Into the Left Atrium Using the Bilateral Transseptal Approach

**DOI:** 10.1016/j.atssr.2025.11.027

**Published:** 2025-12-19

**Authors:** Kaito Yano, Tsutomu Ito, Keisuke Asakura, Daiki Harada, Yorihiko Matsumoto, Yu Okubo, Kyohei Masai, Kaoru Kaseda, Yutaka Kurebayashi, Hideyuki Shimizu

**Affiliations:** 1 Department of Thoracic Surgery, Keio University School of Medicine, Tokyo, Japan; 2 Department of Cardiovascular Surgery, Keio University School of Medicine, Tokyo, Japan; 3 Department of Pathology, Keio University School of Medicine, Tokyo, Japan

## Abstract

Lung tumor with polypoid extension into the left atrium (LA) poses a high systemic tumor embolism risk. We report a rare case of a woman with pulmonary metastasis from a retroperitoneal leiomyosarcoma extending into the LA as a polypoid mass through the left superior pulmonary vein. To minimize the risk of fatal embolization, complete resection was performed under cardiopulmonary bypass using a bilateral transseptal approach, enabling excellent visualization and precise intracardiac tumor resection before left upper lobectomy. She has remained recurrence free for 1 year. This approach safely manages lung tumors with polypoid LA invasion while mitigating catastrophic tumor embolism risk.

Surgical resection, including partial left atrium (LA) excision, may be considered for pulmonary tumors extending into the LA, when deemed feasible.^1^ In such cases, the foremost concern is tumor embolism prevention, which necessitates meticulous preoperative planning and intraoperative caution. Here, we report a rare case of a left upper lobe (LUL) metastatic lung tumor exhibiting polypoid extension into the LA, which was successfully resected using cardiopulmonary bypass (CPB) to minimize embolic complication risk.

A woman in her 60s previously underwent resection of a 70-mm retroperitoneal leiomyosarcoma. Bilateral pulmonary tumors were incidentally detected on computed tomography (CT) 5 years after surgery, prompting her referral to our department. CT revealed a 5-cm and 2.5-cm lesion each in the LUL and the right lower lobe (RLL), respectively (Figure 1). The LUL lesion extended into the LA through the left superior pulmonary vein (PV) in a polypoid manner. Positron emission tomography-CT demonstrated high uptake in both lesions.

**Figure 1:**
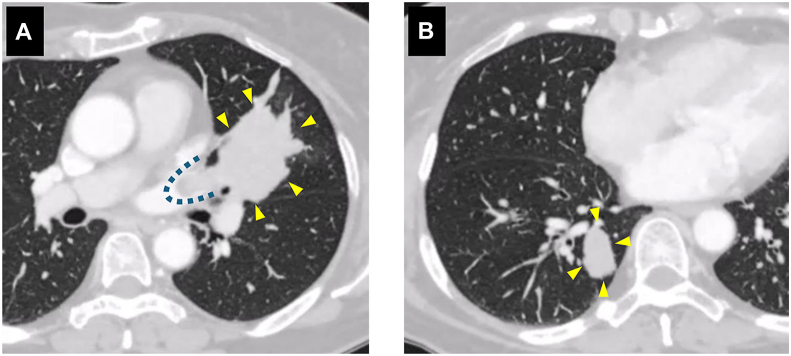
Computed tomography revealed (A) a 5-cm mass in the left upper lobe (yellow arrowheads), with suspected polypoid extension into the left atrium through the left superior pulmonary vein (blue dots), and (B) a 2.5-cm nodule in the right lower lobe (yellow arrowheads).

A clinical diagnosis of bilateral pulmonary metastases from leiomyosarcoma was established. Considering the limited efficacy of systemic therapy, a local treatment strategy was selected. LUL lesion resection with LA extension was prioritized, followed by second-stage treatment of the RLL lesion. Owing to the risk of tumor embolism, surgery was planned under CPB using a bilateral transseptal approach, which entails an incision of the right atrial wall and interatrial septum to access the LA.

Surgical access was achieved through a median sternotomy with left anterolateral extension at the fourth left intercostal space. After taping the ascending aorta and superior vena cava, cannulation was performed, with arterial return through the ascending aorta and venous drainage through the superior vena cava and inferior vena cava.

CPB and cardiac arrest were initiated, and the right atrium and interatrial septum were incised to allow direct visualization of the tumor within the LA (Figure 2). After the LA wall was incised and the pulmonary vein divided, the atrial wall was closed using a direct suture. Moreover, a left upper lobectomy was performed under CPB. After hemostasis, the CPB was weaned off, and surgery was completed. The total operative time, CPB time, and aortic cross-clamp time were 5 hours and 32 minutes, 2 hours and 21 minutes, and 1 hour and 49 minutes, respectively. The operative video is available as a supplemental file (Video). Histopathologic examination confirmed a metastatic leiomyosarcoma (Figure 3).

**Figure 2:**
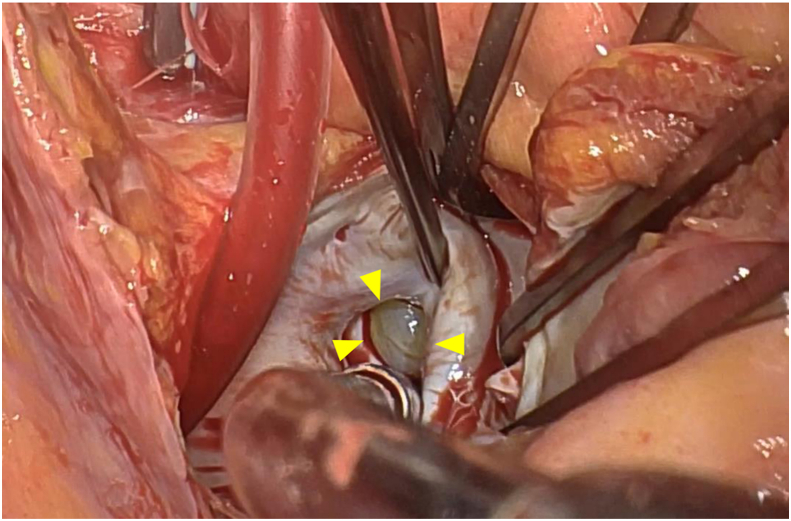
An intraoperative view after incision of the right atrium and the interatrial septum allows for direct visualization of the tumor within the left atrium. The polypoid tumor is seen extending from the left superior pulmonary vein into the left atrium. The yellow arrowheads indicate the polypoid tumor.

**Figure 3:**
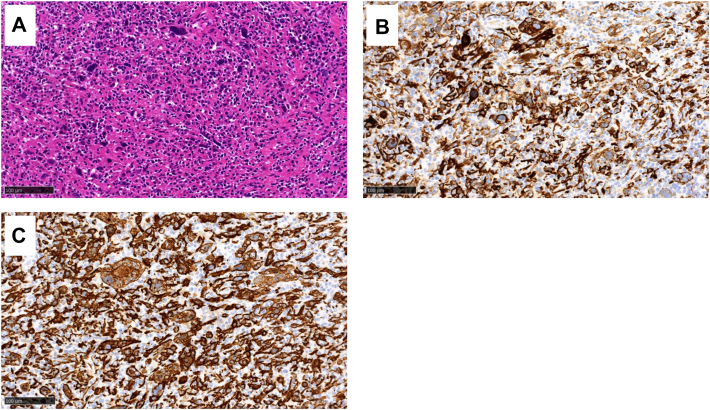
Histopathologic findings of the resected specimen. (A) Hematoxylin and eosin staining shows a proliferation of spindle-shaped cells, with prominent lymphocytic infiltration (original magnification ×100). (B) Immunohistochemical staining demonstrates that the tumor cells are positive for H-caldesmon (original magnification ×100). (C) Immunohistochemical staining shows that the tumor cells are positive for α-smooth muscle actin (original magnification ×100).

Although the postoperative left-sided pleuritis required drainage and antibiotic therapy, the patient recovered well and was discharged on postoperative day 25. The RLL lesion, located in a region unsuitable for wedge resection, was not amenable to anatomical resection because of the limited pulmonary reserve. Stereotactic body radiotherapy (50 Gy) was administered 1 month postoperatively. One year postoperatively, the patient has remained recurrence free.

## Comment

Pulmonary tumors extending into the LA can be categorized into 2 types: direct invasion from the primary tumor or lymph nodes and polypoid intracardiac extension through the PV. In the former, LA resection is performed by applying a clamp proximal to the tumor. Notably, mobilization of the lungs to advance the PV may facilitate safe clamp placement. Moreover, a series of 15 patients with primary lung cancer invading the LA exhibited favorable outcomes when clamping techniques were used.^2^ Nonetheless, clamping becomes technically unfeasible when the tumor invades deep into the LA, necessitating CPB-assisted resection.^3^

When a tumor extends into the LA in a polypoid manner, surgical manipulation must be performed with extreme caution^2^ because of the high risk of dislodging tumor fragments, potentially leading to a systemic “showering” of emboli,^4^ thereby requiring minimal handling of the mass.^5^ Importantly, direct palpation should be avoided because of embolic event risk, and intraoperative echocardiography is recommended to delineate the tumor margins. Because polypoid tumors carry a high risk of fatal embolization during clamping, CPB-assisted resection should be considered.

For non-small cell lung cancer invading the LA, surgical resection using CPB facilitates safe and wide-ranging complete resection, thereby offering a chance for cure or long-term survival.^3^^,^^5^ Despite the risks of bleeding, thromboembolism, hematogenous dissemination of tumor cells, and infection,^6^ this strategy represents a crucial therapeutic option for achieving curative outcomes and ensuring long-term survival. Nevertheless, the appropriateness of such an aggressive surgical approach for metastatic lung tumors, which carry a high recurrence risk, remains controversial. In the present case, however, the tumor was a sarcoma resistant to chemotherapy and radiotherapy with a potential sudden death risk owing to tumor embolism. Therefore, among local treatment options, preceding with surgery on the left side was prioritized over alternatives such as bilateral radiotherapy.

For tumors located within the LA, the bilateral transseptal approach, which entails an incision of the right atrial wall and interatrial septum to access the LA, has been reported as a useful surgical technique.^7^^,^^8^ This method offers excellent LA anterior exposure without cardiac displacement, minimizes tumor dissemination risk, and enables the precise assessment of LA wall invasion. In our case, this approach allowed accurate leading edge of the tumor evaluation, enabling complete LA resection before safely proceeding to lobectomy.

The primary learning point is that this approach is considered useful not only for tumors originating within the LA but also for those that extend polypoids into the LA through the PV from the lung, enabling safe resection while minimizing embolism risk. Conversely, a critical pitfall to consider is that this approach carries an inherent postoperative arrhythmia risk owing to potential injury to the sinoatrial and atrioventricular nodes, warranting meticulous surgical caution.

In conclusion, surgical resection using a bilateral transseptal approach can effectively treat metastatic pulmonary tumors with polypoid extension into the LA through the PV, offering oncologic control while minimizing catastrophic tumor embolism risk.
